# Expression of aurora kinase A correlates with the Wnt‐modulator RACGAP1 in gastric cancer

**DOI:** 10.1002/cam4.610

**Published:** 2016-01-18

**Authors:** Jan Bornschein, Jessica Nielitz, Ignat Drozdov, Michael Selgrad, Thomas Wex, Doerthe Jechorek, Alexander Link, Michael Vieth, Peter Malfertheiner

**Affiliations:** ^1^ Department of Gastroenterology, Hepatology and Infectious DiseasesOtto‐von‐Guericke UniversityLeipziger Str. 4439120MagdeburgGermany; ^2^ Department of Computational BiologyBering Limited80 Third Cross RoadTwickenhamTW2 5EAUnited Kingdom; ^3^ Department of Molecular GeneticsMedical Laboratory for Clinical Chemistry, Microbiology and Infectious DiseasesAm Neustädter Feld 4739124MagdeburgGermany; ^4^ Institute of PathologyOtto‐von‐Guericke UniversityLeipziger Str. 4439120MagdeburgGermany; ^5^ Institute of PathologyKlinikum Bayreuth GmbHPreuschwitzer Str. 10195445BayreuthGermany

**Keywords:** AURKA, CDKN1A, gastric cancer, RACGAP1, Wnt

## Abstract

Canonical Wnt signaling is involved in gastric carcinogenesis. The aim of this study was to identify the link between Wnt signaling and aurora kinase A (AURKA), a target for the treatment of gastrointestinal cancers. Publicly available microarray data were used to identify phenotype‐specific protein–protein interaction (PPI) subnetworks. The in silico analysis revealed a gastric cancer‐specific PPI subnetwork consisting of 2745 proteins and 50,935 interactions. We focused on the link of AURKA to a Wnt‐specific interaction module consisting of 92 proteins. There was a direct association of AURKA with Rac GTPase‐activating protein 1 (RACGAP1), as well as with CTNBB1 (*β*‐catenin) and CDKN1A as second‐order interactors. Differential expression analysis revealed a significant downregulation of both AURKA and RACGAP1 in gastric cancer compared to noncancer controls. Biopsies from a prospective cohort of 56 patients with gastric cancer (32 intestinal type, 24 diffuse type) and 20 noncancer controls were used for validation of the identified targets. The RT‐PCR data confirmed a strong correlation of AURKA and RACGAP1 gene expression both in the tumor, the tumor‐adjacent and the tumor‐distant mucosa. RACGAP1 in the tumor was also associated with CTNBB1 expression, and inversely associated with CDKN1A gene expression. Immunohistochemistry confirmed expression of the RACGAP1 protein in gastric cancer and the tumor‐adjacent mucosa. RACGAP1 expression was not associated with tumor stage, grading, Lauren type, *Helicobacter pylori* infection, or age. In conclusion, AURKA is directly associated with the expression of RACGAP1, a modulator of the canonical Wnt signaling pathway.

## Introduction

As there are no established programs for screening or early detection in most parts of the world, gastric cancer is usually diagnosed at an advanced stage when only palliative treatment modalities can be offered. Palliative treatment is limited to systemic chemotherapy with low response rates and poor prognosis 1. The recent advent of targeted therapies has had only little impact on the prognosis of gastric cancer, still showing an overall median survival of less than 2 years 2, 3. Modern high‐throughput techniques and further system biological in silico approaches have been applied to identify promising targets in signaling pathways relevant to gastric cancer development. Targeting the Wnt/*β*‐catenin pathway has been one focus of interest in the past years and interference with this cascade has been attempted for treatment of several malignancies, with only minor success thus far 4. Free *β*‐catenin is a main target of canonical Wnt signaling, and its aberrant expression has been described mainly in gastric cancer of the intestinal type 5, 6. This seems to be most relevant in the invasive front of early cancers, whereas adjacent noncancerous mucosa and premalignant lesions do not show alterations in *β*‐catenin expression 6.

Aurora kinase A (AURKA) has been shown to be involved in Wnt‐signaling by stabilizing *β*‐catenin 7, 8, and inhibition of AURKA attenuates canonical Wnt signaling 7. *AURKA* is expressed in up to 50% of gastrointestinal cancers and has been reported as a negative prognostic indicator for esophageal adenocarcinoma and gastric cancer 9, 10. *AURKA* gene expression can also be upregulated in gastric inflammation and in premalignant lesions such as intestinal metaplasia (IM) of the gastric mucosa 11, 12. AURKA interferes with several signaling pathways at different levels including NF*κ*B‐related signals by direct phosphorylation of I*κ*B, or upstream interference with GSK‐3*β* 12, 13. Thus, inhibition of AURKA is under evaluation for its therapeutic potential. AURKA inhibition in pediatric medulloblastomas and gliomas enhances chemosensibility via Wnt inhibition 7, 14. In vitro treatment of colorectal cancer cells with the AURKA inhibitor hesperidin showed various effects including enhancement of p21 function and restoration of GSK‐3*β* function, resulting in prevention of *β*‐catenin accumulation in the cell nucleus 15.

In this study, we aimed to carry out an unbiased in silico assessment of publicly available microarray data of gastric cancer gene expression to identify gastric cancer specific protein–protein interaction (PPI) subnetworks. A strong and unexpected association emerged between AURKA and the Wnt modulator Rac GTPase‐activating protein 1 (RACGAP1) in a phenotype‐specific gastric cancer signaling network, that we validated by quantitative RT‐PCR in an independent, prospective cohort of gastric cancer patients. Furthermore, we validated the link to Wnt‐related signaling by evaluation of the related gene expression of *β*‐catenin (*CTNNB1*; main target of the cascade) as well as p21 (*CDKN1A*: cyclin‐dependent kinase inhibitor 1A; intermediate downstream target) 9, 16. This is the first evidence of a direct link of AURKA and Wnt signaling via RACGAP1 in gastric cancer.

## Materials and Methods

### Gene expression analysis

The ArrayExpress database 17 was accessed to identify a comprehensive gastric cancer dataset. We selected a dataset of *n* = 12 gastric cancer samples, *n* = 12 adjacent normal mucosal samples, and *n* = 3 nonadjacent normal mucosal samples 18 (ArrayExpression identifer: GSE19826). Raw CEL files were processed using an online pipeline implemented in R language (freely accessible at www.beringresearch.com). The data were subject to a standardized and strict preprocessing approach in order to facilitate reliable phenotype detection in subsequent analyses:

1. *Present/Absent call detection*. Probesets present in fewer than 50% of all samples were removed 19.
2. Robust microarray average (RMA) normalization.
3. *Array outlier detection*. The *arrayQualityMetrics* 20 Bioconductor package was used to identify and remove outlying samples.
4. *Probe annotation*. Probe identifiers (IDs) were mapped to Entrez Gene IDs (accessed January 10, 2015). In cases where multiple probes mapped to the same Entrez ID, the average probe intensity was calculated. Probes without an Entrez record were removed from analysis.
5. *Differential expression analysis*. Comparison statistics between cases and controls was calculated using the *limma* package 21.

### Protein–protein interaction network analysis and subgraph extraction

STRING database (version 9.1) was used as a source of PPIs 22. STRING combines heterogeneous interactions (e.g., experimentally determined interactions, gene neighborhood data, or data acquired via text mining) into a single object that can be queried via the *STRINGdb* Bioconductor package.

In STRING, all interactions were assigned with a confidence score ranging from 0 to 1. In order to direct the analysis toward a highly confident network, interactions with a score <0.850 were excluded, yielding 11,442 nodes and 164,719 interactions. Subsequently, all genes on the processed microarray were mapped onto nodes of the PPI network; nodes without a direct mapping were removed.

The phenotype‐specific gastric cancer network was identified using a genetic algorithm‐driven approach. Briefly, all network nodes were assigned a weight of $-{\text{log}}_{10p}$, where *P* is the unadjusted *P*‐value obtained from differential expression analysis. The algorithm then searches for the best combination of nodes to yield the most highly weighted subnetwork 23. The advantage of this method is that it does not require stringent thresholding parameters, such as a *P‐*value or fold change, thereby eliminating the associated interpretation bias 24.

### Patient cohort validation study

In total, 56 patients with gastric cancer (32 intestinal, 24 diffuse type) and 20 noncancer controls were included in the study. Exclusion criteria were: presence of malignant diseases other than gastric cancer, chronic inflammatory diseases, malabsorptive diseases, abnormal coagulation parameters, intake of immunosuppressive medication, severe renal or hepatic impairment or any contraindication against upper gastrointestinal endoscopy. Additional exclusion criteria for the control group were peptic ulcers disease, erosive lesions in the upper gastrointestinal tract, and history of reflux disease. None of the patients with gastric cancer had chemotherapy, radiotherapy, or surgical intervention prior to endoscopy. Table 1 shows detailed demographic data of the patients with gastric cancer. The control group comprised 20 patients (mean age 26.8 ± 5.9 years) with neither malignant disease, nor *H. pylori* infection or gastroesophageal reflux disease. The study was in line with the Declaration of Helsinki and approved by the local ethics committee and government authorities (registered as 132/01 and 34/08). Written informed consent was obtained from all subjects.

**Table 1:** Demographic and histopathological data of the gastric cancer patients

	Intestinal (*n* = 32)	Diffuse (*n* = 24)	Total (*n* = 56)	*P*‐value
Age (years; median, range)*	72.5 (46.6–94.3)	61.4 (30.3–78.0)	67.8 (30.3–94.3)	0.001
Sex (male)*	22 (68.8%)	9 (37.5%)	31 (55.4%)	0.03
Location of the tumor				
Cardia	8 (25.0%)	3 (12.5%)	11 (19.6%)	(0.180)
Corpus	13 (40.6%)	16 (66.7%)	29 (51.8%)	
Antrum	11 (34.4%)	5 (20.8%)	16 (28.6%)	
Proximal	12 (37.5%)	9 (37.5%)	21 (37.5%)	(1.000)
Distal	20 (62.5%)	15 (62.5%)	35 (62.5%)	
Degree of differentiation*				
Well	5 (15.6%)	0 (0.0%)	5 (8.9%)	0.001
Moderate	14 (43.8%)	3 (12.5%)	17 (30.4%)	
Poor	13 (40.8%)	21 (87.5%)	34 (60.7%)	
T‐stage^a^				
T1	6 (24.0%)	5 (21.7%)	11 (22.9%)	(0.542)
T2	3 (12.0%)	3 (13.0%)	6 (12.5%)	
T3	15 (60.0)	11 (47.8%)	26 (54.2%)	
T4	1 (4.0%)	4 (17.4%)	5 (10.4%)	
N‐stage^a^ (positive)	12 (54.4%)	16 (76.2%)	28 (65.1%)	(0.203)
M‐stage^a^ (M1)	9 (47.4%)	9 (47.4%)	18 (47.4%)	(1.000)
*Helicobacter pylori* (positive)	26 (81.2%)	15 (62.5%)	41 (73.2%)	(0.138)
IM (present)	23 (71.9%)	11 (45.8%)	34 (60.7%)	(0.059)
Atrophy (present)	12 (37.5%)	4 (16.7%)	16 (28.6%)	(0.135)

Parameters were compared according to Laurén type (*t*‐test for age, Fisher's exact test for categorical data, significance for *P *< 0.05). Significant categories are marked by an asterisk. IM, intestinal metaplasia.

a Data for TNM were incomplete; percentages are given for the available numbers.

### Upper gastrointestinal endoscopy and histopathological assessment

All patients and controls underwent upper gastrointestinal endoscopy. In patients with gastric cancer, location of the tumor was endoscopically classified according to the position of the main tumor mass as described previously 25. Biopsies were taken from each the tumor itself, tumor‐adjacent mucosa (within 1 cm from the macroscopic tumor margin), and from tumor‐distant mucosa (at least 3 cm distant from the macroscopic tumor margin). For the noncancer controls, biopsies were taken according to the protocol as defined by the updated Sydney classification 26. One sample from each localization was snap‐frozen in liquid nitrogen and stored at −80°C until usage for molecular analysis. Another sample was formalin‐fixed for routine histopathological assessment. One section was stained each with hematoxylin and eosin, and modified Giemsa for detection of *H. pylori*. *Helicobacter pylori* infection was additionally determined by rapid urease test (HUT^®^; Astra‐Zeneca, Wedel, Germany) and serology. *Helicobacter pylori* status was regarded as positive if one test modality was positive. The typing and grading of gastritis including IM and glandular atrophy was performed according to the updated Sydney classification. All parameters were semiquantitatively scored as either 0 (absent), 1 (mild), 2 (moderate), or 3 (severe).

### Extraction of total RNA and quantitative PCR analysis

RNA extraction was performed using the Qiagen RNeasy Plus Universal Mini Kit 73404 (Qiagen, Hilden, Germany) according to the single step method (27). cDNA synthesis was performed for 45 min at 42°C on a Thermomixer (Eppendorf, Hamburg, Germany) using 2000 ng RNA in 25.4 *μ*L RNAse‐free water and 14.6 *μ*L mastermix (1.0 *μ*L Recombinant‐Rnasin Ribonuclease Inhibitor, 8.0 *μ*L AMV‐RT 5x reaction buffer, 2.0 *μ*L AMV reverse transcriptase (each Promega, Mannheim, Germany); 1.6 *μ*L dNTP‐mix (10 mmol/L), Peqlab Biotechnologie GmbH, Erlangen, Germany; 2.0 *μ*L Random‐Primer (0.04 A260U/*μ*L), Roche Diagnostics, Mannheim, Germany). Inactivation of the reverse transcriptase was achieved by heating to 95°C for 5 min. Quantitative RT‐PCR was performed using a BIO‐RAD CFX96 Touch^TM^ Real‐Time PCR Detection System (BioRad Laboratories GmbH, Munich, Germany). The reaction mixture consisted of 15 *μ*L 2x Quanti‐Tect RSYBR‐Green Mastermix R (Qiagen), 13.4 *μ*L RNase‐free water, 0.2 *μ*L of both forward (fw) and reverse (rev) primer for each gene, and 1.2 *μ*L cDNA (40 cycles, annealing temperature 60°C for 30 sec, incubation 95°C, elongation 72° for 30 sec; annealing for RACGAP1 at 54°C, for *β‐actin* and DKK2 58°C).

The following primers were used for the qRT‐PCR analysis: *AURKA* (fw: 5′‐ttctggaatatgcaccacttg‐3′; rev: 5′‐aagctctccagctgatccaa‐3′), *RACGAP1* (fw: 5′‐tctcaacagaggccaaccatcc‐3′; rev: 5′‐actgcagagccaatggaacgag‐3′), *CTNNB1* (fw: 5′‐catgccatcctgcgtctgcacc‐3′; rev: 5′‐acatggtggtgccgccagaca‐3′), *CDKN1A* (fw: 5′‐gctgcgttcacaggtgtttctg‐3′; rev: 5′‐tggtgtctcggtgacaaagtcg‐3′), *β‐actin* (fw: 5′‐catgccatcctgcgtctggacc‐3′, rev: 5′‐acatggtggtgccgccagaca‐3′). Gel electrophoresis was performed for verification of the size of the RT‐PCR product for each gene. Final data represent the ratio of each gene to *β*‐actin transcript given as arbitrary units (a.u.).

### Immunohistochemistry

Three‐micrometer sections from formalin‐fixed and paraffin‐embedded tissue were incubated overnight at 60°C followed by stepwise rinsing in Xylol and ethanol dilution series. Slides were heated for 64 min 100°C in 0.01 mol/L EDTA buffer using a steam‐pressure chamber. The primary polyclonal Anti‐RACGAP1 goat antibody was applied at a dilution of 1:200 (reference number: ab2270; Abcam, Cambridge, UK) and incubated at 4°C for 3 h before rinsing with distilled water and reaction buffer. The secondary antibody was applied for 30 min at room temperature. For automated staining, the NexES IHC staining module (Roche Medical Systems, Inc., Munich, Germany), the iVIEW DAB Detection Kit (Ventana Medical Systems, Inc., Munich, Germany) and the indirect biotin–streptavidin method were used before counterstaining with Haemalaun solution. Specificity of the staining was tested by selective substitution of the primary antibody by nonimmunogenic serum.

Semi‐quantitative evaluation of the staining reaction was undertaken using the modified immune‐reactivity score (IRS) 28. The partition of positively stained cells (PP: 0–100%, represented by scores 0–10 with 0 = 0%, 1 = 10%, and 10 = 100%) was multiplied with the staining intensity (SI: 0–3; 0 = negative, 1 = weak, 2 = moderate, 3 = strong reaction), resulting in a score ranging from 0 to 30. Scoring has been applied separately for each patient for the tumor center represented by the main bulk of malignant cell conglomeration, and for the invasive front, represented by scattered malignant cells at the border of the adjacent tissue structures, mostly stromal compartments.

### Statistical analysis

Data were analyzed using SPSS 21.0 (IBM SPSS Statistics, Chicago, IL); graphs were generated with GraphPad Prism 6.0 (GraphPad Software Inc., San Diego, CA). Categorical data were compared applying Fisher's exact test. The distribution of age between groups was assessed by Student's *t*‐test. For statistical analyses of gene expression values among subgroups, nonparametric tests were used in order to take account for possibly skewed distributions. For comparison with controls, Kruskal–Wallis test was applied for primary global analysis, followed by the Mann–Whitney *U*‐test for post hoc analysis in case of positive results. For matched pair‐wise comparison of the different sample sites in each patient (tumor, tumor‐adjacent, tumor‐distant), Wilcoxon's signed rank test was applied as post hoc analysis after positive result of the nonparametric Friedman test for *k*‐related samples. Correlation analysis was done by Spearman's rank correlation test. All tests were calculated considering a two‐sided significance level of *P *<* *0.05. Bonferroni's correction was applied in case of multiple comparisons.

## Results

### In silico analysis and target identification

**Figure 1:**
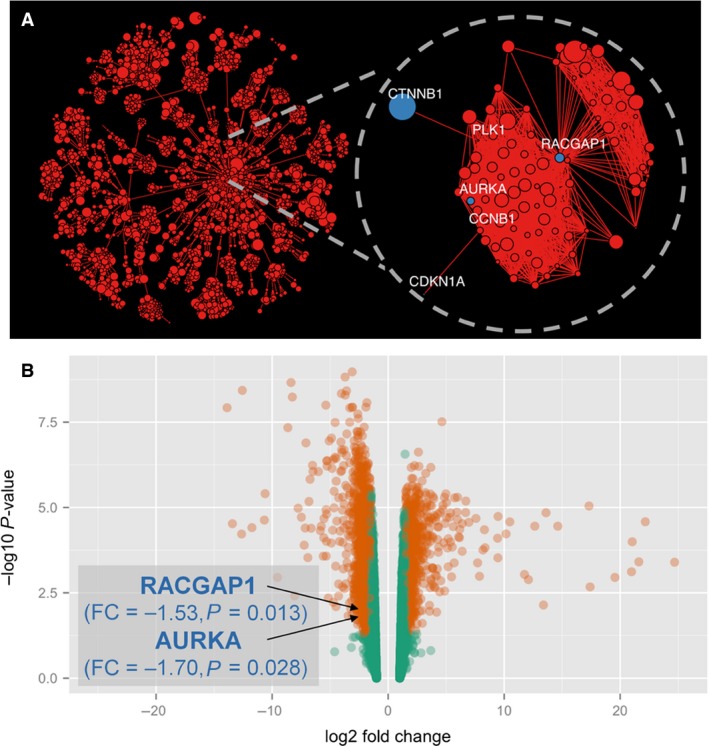
Protein network analysis of a gastric cancer gene expression set. (A) Gastric cancer‐specific subnetwork consisting of 2745 proteins and 50,935 interactions as revealed by STRING PPI analysis of the microarray data of 12 gastric cancer samples, 12 matched adjacent nontumorous mucosa samples, and three healthy gastric mucosa controls. The inlet shows a Wnt‐associated module consisting of 92 proteins. *RACGAP1* is directly linked to *AURKA*, while Wnt‐signaling targets *CTNBB1* and *CDKN1A* are second‐order interactors, linked via *CCNB1* and *PLK1*, respectively. (B) Differential expression analysis revealed a statistically significant downregulation of *RACGAP1* (fold change = −1.53, *P* = 0.013) and *AURKA* (fold change = −1.70, *P* = 0.028) in cancer tissue compared to nontumorous mucosa. Targets with statistically significant differential expression are displayed in orange, otherwise in green.

Differential expression analysis was undertaken on 16,120 probesets spanning 27 samples. All obtained *P*‐values were mapped to the STRING PPI network in order to identify a maximally scoring subnetwork. The resulting gastric cancer‐specific subnetwork consisted of 2745 proteins and 50,935 interactions (Fig. 1A). Within this subnetwork, we identified a strong Wnt‐associated module, consisting of 92 proteins (Fig. 1A inset), underpinned by *RACGAP1*. Interestingly, *AURKA* expression was directly linked with *RACGAP1* in this network, while *CTNNB1* (*β*‐catenin) and *CDKN1A* were second‐order interactors, linked with *RACGAP1* via *CCNB1* (cyclin B1) and *PLK1* (polo‐like kinase 1), respectively. Differential expression analysis confirmed statistically significant downregulation of *RACGAP1* (fold change = −1.53, *P *=* *0.013) and *AURKA* (fold change = −1.70, *P *=* *0.028; Fig. 1B).

### Gene expression analysis for AURKA

**Figure 2:**
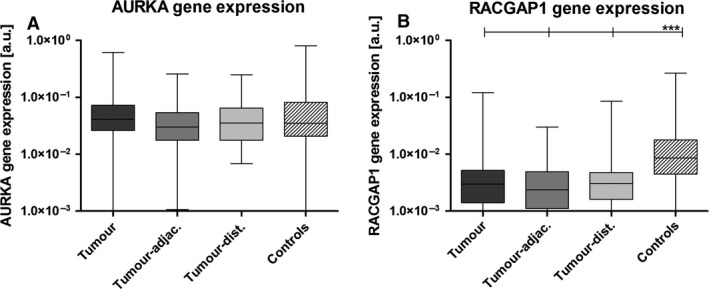
Gene expression of AURKA and RACGAP1 in cancer patients and controls. Gene expression analysis of (A) *AURKA* and (B) *RACGAP1* on mRNA level for samples from tumor, tumor‐adjacent, and tumor‐distant mucosa for cancer patients as well as for gastric biopsies from noncancer controls. Cancer patients showed a homogeneous expression in the tumor and the nontumorous mucosa. For RACGAP1, the gene expression was significantly lower compared to controls. The boxplots show mean, 25th, and 75th percentiles as well as minimum and maximum of the mRNA content in arbitrary units [a.u.]. Statistically significant differences are marked with asterisks (*P < 0.05, **P < 0.01, ***P < 0.001).

These findings are validated using RT‐PCR in an independent cohort of 56 patients with gastric cancer and 20 noncancer controls. In patients with gastric cancer, *AURKA* gene expression was detected in all of the tumor samples, and also in all tumor‐adjacent and tumor‐distant mucosa biopsies. The gene expression in cancer samples was overall similar to the expression in our control group (*P *=* *0.399; Fig. 2A). There was no statistically significant difference of the *AURKA* gene expression between tumor, tumor‐adjacent, and tumor‐distant tissue (*P *=* *0.476; Fig. 2A). Furthermore, there was no difference in *AURKA* gene expression between cancers of the intestinal and the diffuse type (*P *=* *0.426) and between cancers of different stage of disease (*P *=* *0.533) or degree of differentiation (*P *=* *0.211). There was also no difference in *AURKA* gene expression in cancers of different location (*P *=* *0.924). *AURKA* was expressed in 95% of the control samples. However, within noncancer controls, the expression of *AURKA* in the antrum was 3.5‐fold higher than in the gastric body (2.23e^−02^ vs. 7.91e^−02^ [a.u.], *P *<* *0.001; Fig. S1A). Therefore, the expression in the antrum samples of controls was higher than in tumor samples (*P *=* *0.001), whereas expression in the body of controls was lower than in tumor samples (*P *=* *0.002; Fig. S1A).

### Gene expression analysis for RACGAP1

Gene expression of *RACGAP1* in gastric cancer samples was significantly lower than in noncancer control biopsies (*P *<* *0.001; Fig. 2B). There was no difference in *RACGAP1* gene expression between tumor, tumor‐adjacent, and tumor‐distant tissue in patients with gastric cancer (*P *=* *0.138; Fig. 2B). There was no difference in *RACGAP1* between tumors of different Laurén type (*P *=* *0.653), of different location within the stomach (*P *=* *0.629), and between cancers of different stage of disease (*P *=* *0.966) or degree of differentiation (*P *=* *0.516). The expression values were independent from *H. pylori* infection status (*P *=* *0.720). Within the control group, gene expression of *RACGAP1* was 2.9‐fold higher in the antrum mucosa of controls when compared with the normal gastric body (4.25e^−03^ vs. 1.25e^−02^ [a.u.], *P *=* *0.011; Fig. S1B). Therefore, expression values in tumor samples were only statistically different when compared with the antrum of controls (*P *<* *0.001), not when compared with normal body (*P *=* *0.1; Fig. S1B).

### Correlation of RACGAP1 gene expression with AURKA

**Figure 3:**
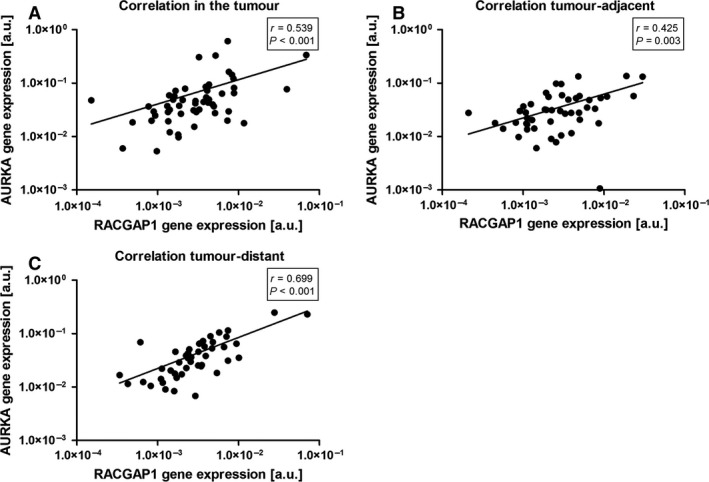
Correlation of AURKA and RACGAP1 gene expression in gastric cancer patients. The mRNA content for the *AURKA* transcript (*y*‐axis) is displayed against the mRNA level for *RACGAP1* (*x*‐axis) in arbitrary units [a.u.] for (A) tumor tissue, (B) tumor‐adjacent mucosa, and (C) tumor distant mucosa. Correlation coefficient (*r*) and *P‐*value according to Spearman's rank correlation test are shown in the box.

There was a positive correlation of *AURKA* with *RACGAP1* gene expression in the tumor (*r *=* *0.539, *P *<* *0.001; Fig. 3A). Similarly, there was a positive association between *AURKA* and *RACGAP1* gene expression in the tumor‐adjacent (*r *=* *0.425, *P *=* *0.003; Fig. 3B) and the tumor‐distant mucosa (*r *=* *0.699, *P *<* *0.001; Fig. 3C). A strong correlation in controls could only be shown for biopsies taken from the antrum (*r *=* *0.775, *P *<* *0.001), not for the gastric body.

### Correlation of RACGAP1 gene expression with Wnt‐related targets

In order to assess the association of *RACGAP1* with Wnt‐related genes, we analyzed the gene expression of *CTNNB1* as the main target of canonical Wnt‐signaling, and *CDKN1A* as intermediate downstream target. In the tumor tissue there was a weak positive correlation of *RACGAP1* gene expression with *CTNNB1* (*r *=* *0.288, *P *=* *0.033; Fig. S2A), as well as an inverse association with *CDKN1A* gene expression (*r *=* *−0.357, *P *=* *0.012; Fig. S2B). In the tumor‐adjacent mucosa, this was confirmed only for *RACGAP1* and *CTNNB1* (*r *=* *0.401, *P *=* *0.003; Fig. S2C). No association could be confirmed in the tumor‐distant mucosa.

### Immunohistochemical staining for RACGAP1 in gastric cancer

**Figure 4:**
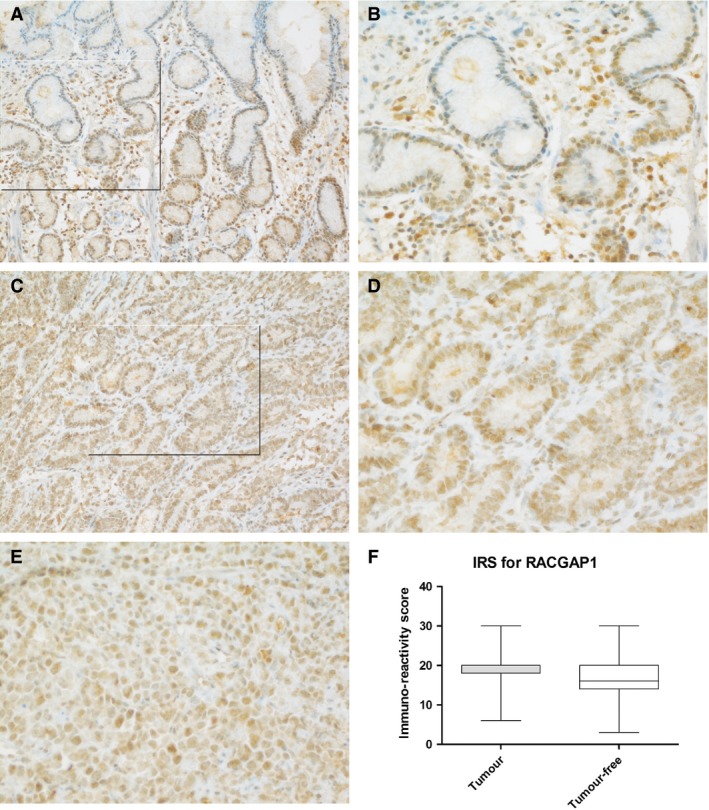
Immunohistochemical staining of RACGAP1 in gastric cancer. Images represent immunohistochemical staining of the RACGAP1 protein in tumor‐distant cancer‐free mucosa (A: ×200, B: ×400) and the tumor center of gastric cancer of the intestinal type (C: ×200, D: ×400), and the diffuse type (E: ×400). The staining pattern in the tumor center shows a homogeneous distribution; in the nonmalignant mucosa, the staining is more intensive at the base of the gastric glands compared to the surface epithelium. The boxplot shows the immune‐reactivity score (IRS) for staining of the tumor and the tumor‐free mucosa (F). There was no statistically significant difference in the staining scores for RACGAP1 between the tumor center and tumor‐distant noncancerous mucosa. The boxplots show mean, 25th, and 75th percentiles as well as minimum and maximum of the mRNA content in arbitrary units [a.u.].

All investigated tumor samples showed mainly nuclear staining of the RACGAP1 protein in at least 30% of the tumor cells. This was also true for the tumor‐free gastric mucosa (Fig. 4). The tumor center shows a more homogeneous distribution of positive staining. In the tumor‐free mucosa, staining at the base of the gastric glands seems to be more pronounced than at the surface epithelium. There was no statistically significant difference in the immune‐reactive staining scores between the tumor and tumor‐free mucosa (*P* =* *0.63; Fig. 4F). There was also no difference between tumors of different Laurén type (*P *=* *0.662), different location (*P *=* *0.832), different T‐stage (*P *=* *0.775), and degree of differentiation (*P *=* *0.641). *Helicobacter pylori* infection status had no influence on RACGAP1 staining in patients with gastric cancer (*P *=* *0.104).

## Discussion

In this study, we confirm a strong association between AURKA and the Wnt modulator RACGAP1*,* as predicted by an in silico graph theoretic approach. To our knowledge, this is the first evidence for RACGAP1 being a link between AURKA and the Wnt signaling cascade.

*AURKA* expression was detected by qRT‐PCR in all of the cancer samples, and there was also homogeneous expression in the noncancerous gastric mucosa of cancer patients. In cancer samples, there was no difference related to the location of the tumor or to any other tumor‐related factor‐like stage or grading. Interestingly, the gastric mucosa of controls showed differential expression of *AURKA*, which was significantly higher in the antrum than in the gastric body. All controls were *H. pylori* negative, but an influence of possible duodenogastric bile reflux and related inflammation in the antrum cannot be ruled out. AURKA is a modulator of several signaling hubs that are involved in procarcinogenic pathways and we could confirm a strong correlation with *RACGAP1* at the gene expression level. RACGAP1 is involved in the regulation of cellular motility and migration by mediating an *α*5*β*1‐dependent switch from Rac to RhoA activation 29. This induces amoeboid movement of cells that then show invasive behavior 30. Silencing of *RACGAP1* in cell lines with endogenous expression inhibits cell migration and invasion 31. RACGAP1 also interferes with the mitotic spindle apparatus, therefore being involved in the regulation of cell proliferation 32. So far, aurora kinase B (AURKB) has been reported to interact with RACGAP1 in solid cancers 31, 33. For AURKA, previous evidence has suggested only an indirect link via AURKA‐mediated STAT3 activation and RACGAP1‐dependent STAT signaling 12, 34. Our PPI network analysis suggests a direct association between AURKA and RACGAP1.

RACGAP1 is expressed by a variety of solid tumors, including breast cancer and hepatocellular carcinoma where it is also a prognostic indicator for early recurrence 31, 35, 38. In our study, the expression of *RACGAP1* in tumor samples was lower compared to noncancer controls, and (similar to *AURKA*) there was no difference in *RACGAP1* gene expression between tumor tissue and nontumorous mucosa in cancer patients. A recent study from Japan showed a higher expression in intestinal type cancers by IHC with the staining scores being higher for the tumor center when compared with the adjacent nontumor tissue 26. Presence of the RACGAP1 protein correlated with age, tumor size, and stage of disease with RACGAP1 expression representing an indicator of poor prognosis. The staining pattern that we observed in our cohort was similar to the results reported in the Japanese study, especially with respect to the distribution of stained cells along the gastric glands in the tumor‐free mucosa. However, we could not confirm a difference between tumor and tumor‐free mucosa in our IHC analysis. An explanation might be that in the Japanese study not only a different immunohistochemistry scoring system was applied, but also only surgical samples were used, whereas we assessed endoscopic biopsies. Furthermore, nearly half of the Japanese patients were diagnosed with stage I cancers including 42% T1 tumors, whereas only 19.6% of our cohort was classified as stage I, and 57.1% had stage III and stage IV disease. There might be a possible field effect on the noncancerous gastric mucosa at the stage of advanced gastric cancer, especially with regard to the role of RACGAP1 regulating invasion and proliferation 26. Additionally, post‐transcriptional modifications need to be taken into account, as both studies revealed discrepancies between the mRNA data and the respective staining scores for the RACGAP1 protein. For neither the IHC nor the PCR data we could confirm a significant association of RACGAP1 expression with tumor stage, tumor location, Laurén type, or *H. pylori* infection status. There have been similar results for different cutoff levels for the IRS. Again, this might be related to the high proportion of advanced stage cancers in our cohort, and a main effect of RACGAP1 at early stages of gastric carcinogenesis cannot be ruled out. The lower expression in gastric cancer samples compared to controls in our gastric cancer cohort is in line with the downregulation of *RACGAP1* that was predicted by the computational analysis (see also Fig. 1B). Contrary to these data in gastric cancer, there was a recent report on upregulation of *RACGAP1* in colorectal cancer with *RACGAP1* being an independent predictive factor for prognosis 39. However, a recent study on colorectal cancers demonstrated that the intracellular localization of the RACGAP1 protein dictates its relevance as prognostic predictor, with only nuclear staining indicating a poor prognosis and cytoplasmic staining actually resulting in a favorable outcome for the patient 40. In our cohort, the pattern of staining was heterogeneous, but mainly showing cytoplasmic staining of RACGAP1 partly with equivocal nuclear expression, a pattern to which an intermediate prognostic relevance has been attributed 38. In addition to this, micro‐array data on the RACGAP1 transcript content in breast cancers have been reported to be only of prognostic relevance in a subgroup of tumors 41. This is yet to be investigated in gastric cancers which are confirmed to be of highly heterogeneous biology 42, 43. Prognostic assessment was not the focus of the present study as we aimed to experimentally demonstrate the in silico link between AURKA and Wnt signaling in gastric cancer at the gene expression level. Our cohort represents a heterogeneous group of patients that underwent the whole spectrum of both palliative (mainly palliative systemic chemotherapy) and curative treatment pathways (surgical or endoscopic resection, partly combined with neoadjuvant or adjuvant systemic treatment) making a stratified analysis of RACGAP1 as prognostic factor for our cohort unfeasible.

In addition to revealing the main connection between AURKA and RACGAP1, the in silico analysis revealed an indirect link of AURKA‐RACGAP1 with both *CTNNB1* (*β*‐catenin) and *CDKN1A* as targets of canonical Wnt signaling, mediated via *PLK1* and *CCNB1*, respectively. *β*‐Catenin itself is involved in the carcinogenesis mainly of intestinal type gastric cancers 5, 6, and a dysregulation of *β*‐catenin expression is related to infection with a CagA‐positive *H. pylori* strain 44, 45. There are conflicting reports concerning the association of *β*‐catenin with stage of disease, tumor differentiation, and the expression in adjacent nonmalignant mucosa 46. There was a weak correlation of *RACGAP1* expression with *CTNNB1* which might have been stronger with an increase in sample size. The association was more evident for the tumor‐adjacent samples which could be due to less dedifferentiation in this part of the gastric mucosa than in the tumor center. In colorectal cancer cells, *β*‐catenin can be activated via the Rac/PAK1 (p21 activating protein 1) cascade by direct phosphorylation of *β*‐catenin by PAK1, which is therefore a link to the intermediate Wnt target p21 (*CDKN1A*) 16. P21 is expressed in 38–68% of gastric cancers and is a positive predictor for survival 47. Especially for Epstein–Barr virus‐related gastric cancer, there is an inverse relation between p21 and *β*‐catenin, which might be mediated by PAK1‐dependent interference with Wnt signaling 48. There was a weak inverse association of p21/*CDKN1A* expression with *RACGAP1*, which again represents the potential link to *AURKA*. Previous studies have demonstrated a reduction in p21 by AURKA induction and enhancement of p21 by treatment with AURKA inhibitors 9, 15. Again, the low correlation coefficient in our data might be due to the limited sample size which is the main limitation of the prospective study cohort we used for validation of the in silico results of this study. It is likely that the signaling cascade between AURKA and p21/*CDKN1A* is modulated by several interactors, so these results need to be interpreted with caution.

In conclusion, this is, to our knowledge, the first study showing an association of AURKA and RACGAP1 in gastric cancer as well as a connection with Wnt‐signaling components. This link needs functional validation to assess RACGAP1 as a novel target that could be introduced in the treatment of gastrointestinal cancers in addition to current approaches using inhibitors of AURKA and Wnt components in clinical trials.

## Conflict of Interest

I. D. is an employee at Bering Limited. The company had no role in design and execution of the current study. Therefore there is no conflict of interest that has to be declared in relation to this work.

## Supporting information


**Figure S1.** Gene expression of AURKA and RACGAP1 in tumor and gastric antrum and body of controls. Gene expression of (A) *AURKA* and (B) *RACGAP1* on mRNA level for samples from the tumor center as well as for gastric biopsies from antrum and body of noncancer controls. For both *AURKA* and *RACGAP1*, gene expression was highest in the antrum of controls. The boxplots show mean, 25th, and 75th percentiles as well as minimum and maximum of the mRNA content in arbitrary units [a.u.]. Statistically significant differences are marked with asterisks (*P < 0.05, **P < 0.01, ***P < 0.001).

**Figure S2.** Correlation of RACGAP1 gene expression with Wnt‐related targets. The mRNA content in tumor tissue for (A) *CTNBB1* and (B) *CDKN1A* is displayed against the mRNA level for *RACGAP1* in arbitrary units [a.u.]. (C) Correlation of *CTNBB1* and *RACGAP1* in the tumor‐adjacent mucosa. Correlation coefficient (*r*) and *P‐*value according to Spearman's rank correlation test are shown in the box.
