# Supplementary figures and images for: Expression of aurora kinase A correlates with the Wnt‐modulator RACGAP1 in gastric cancer
Source: Cancer Med. 2016 Jan 18;5(3):516–26. doi: 10.1002/cam4.610 (PMC4799948; doi:10.1002/cam4.610)

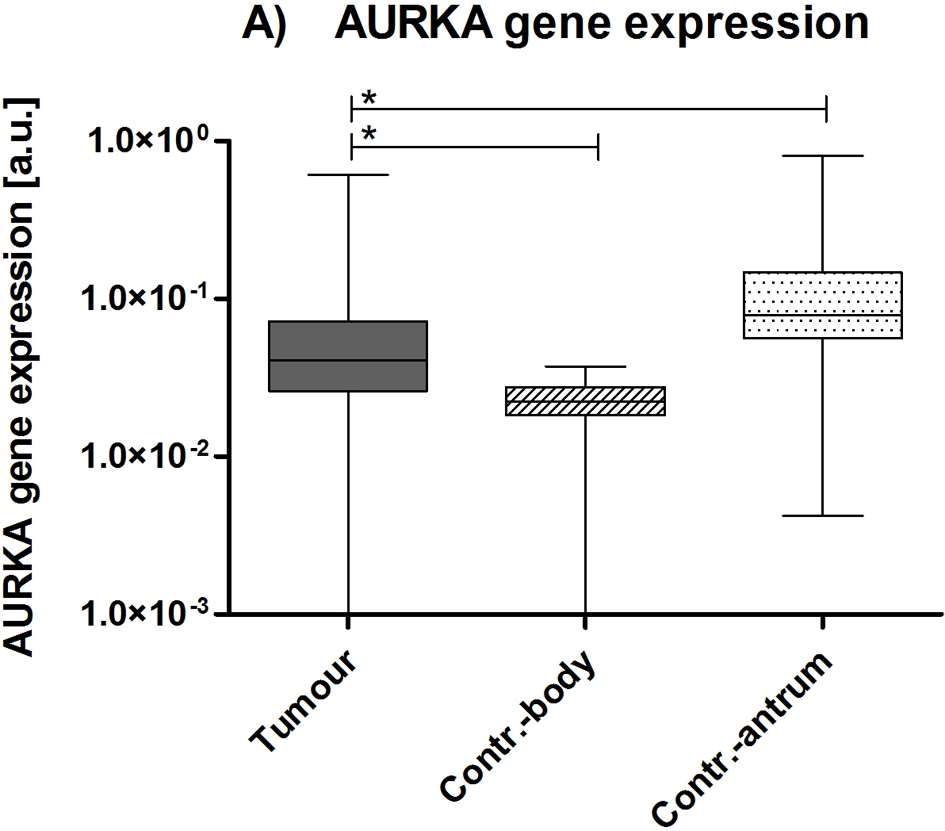

Supplement: Supplementary file 1 — Figure S1. Gene expression of AURKA and RACGAP1 in tumor and gastric antrum and body of controls. Gene expression of (A) AURKA and (B) RACGAP1 on mRNA level for samples from the tumor center as well as for gastric biopsies from antrum and body of noncancer controls. For both AURKA and RACGAP1, gene expression was highest in the antrum of controls. The boxplots show mean, 25th, and 75th percentiles as well as minimum and maximum of the mRNA content in arbitrary units [a.u.]. Statistically significant differences are marked with asterisks (*P < 0.05, **P < 0.01, ***P < 0.001). [file CAM4-5-516-s001.tif]

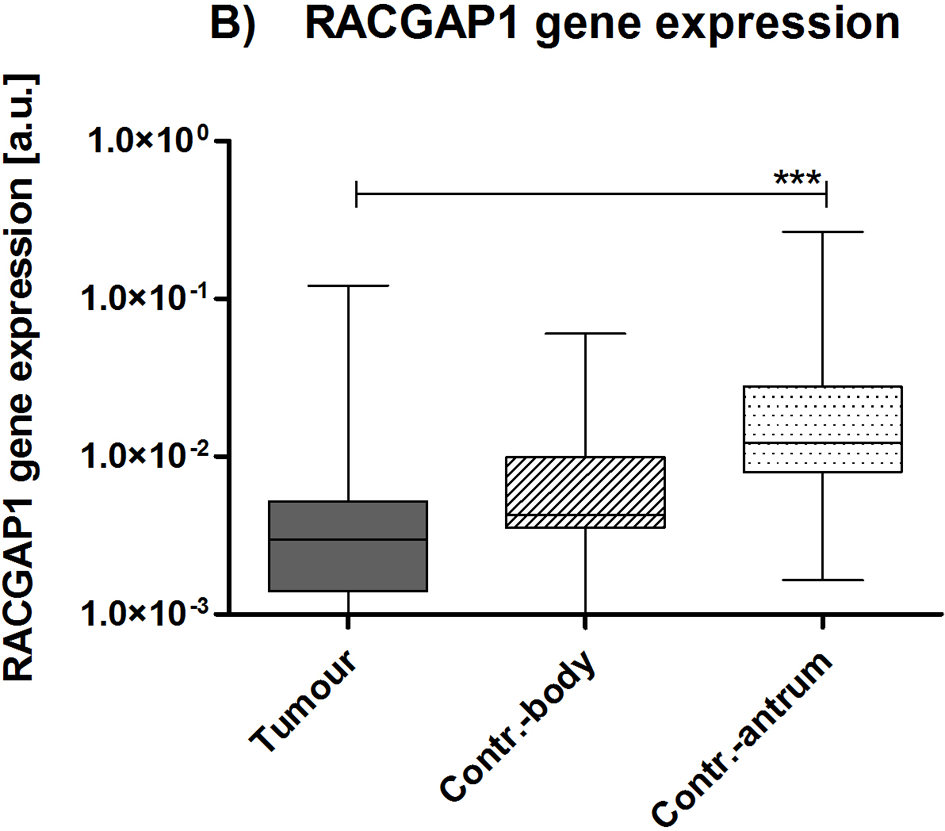

Supplement: Supplementary file 2 [file CAM4-5-516-s002.tif]

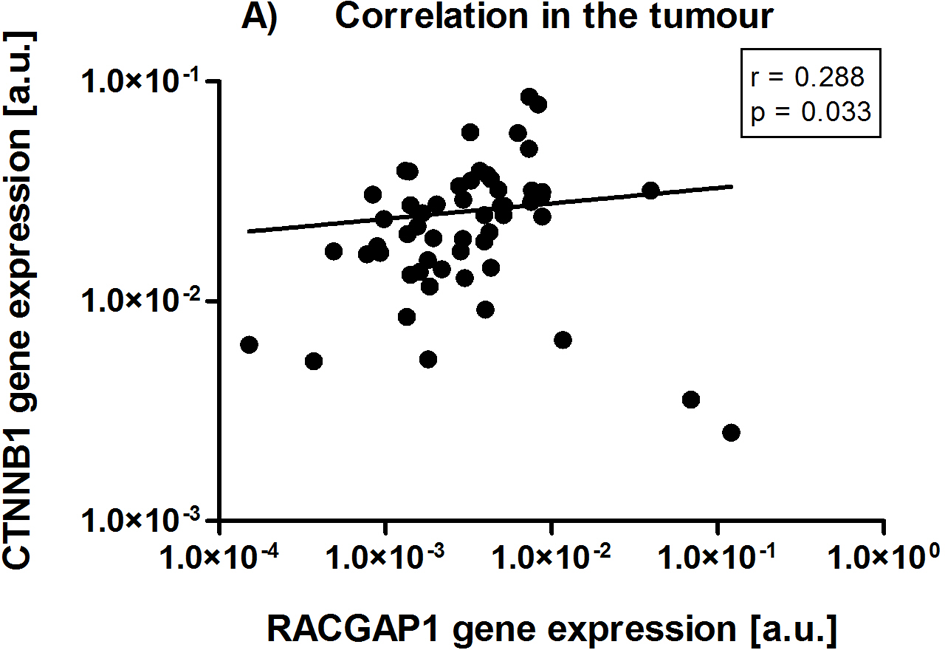

Supplement: Supplementary file 3 — Figure S2. Correlation of RACGAP1 gene expression with Wnt‐related targets. The mRNA content in tumor tissue for (A) CTNBB1 and (B) CDKN1A is displayed against the mRNA level for RACGAP1 in arbitrary units [a.u.]. (C) Correlation of CTNBB1 and RACGAP1 in the tumor‐adjacent mucosa. Correlation coefficient (r) and P‐value according to Spearman's rank correlation test are shown in the box. [file CAM4-5-516-s003.tif]

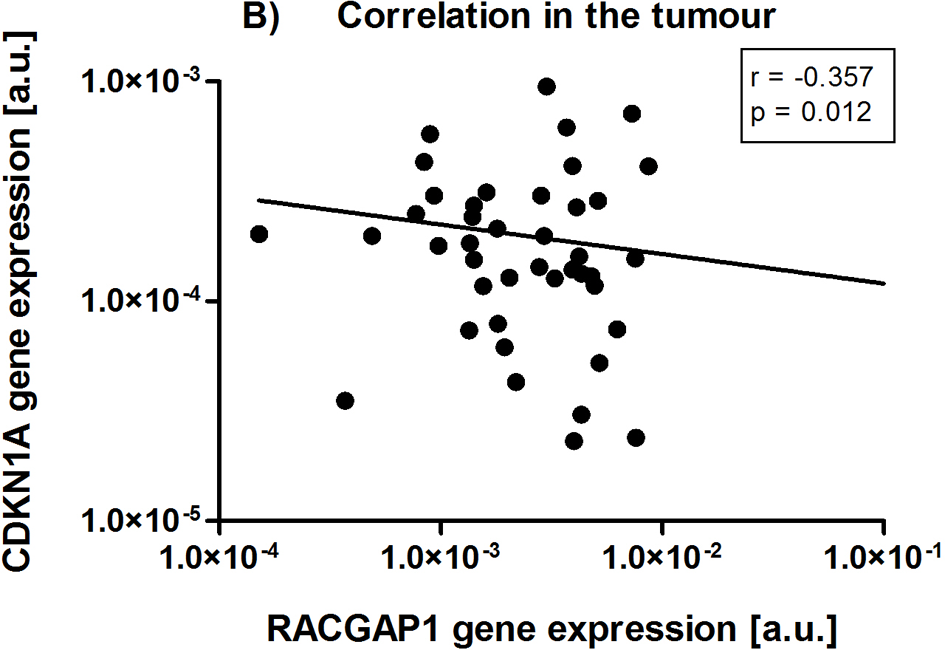

Supplement: Supplementary file 4 [file CAM4-5-516-s004.tif]

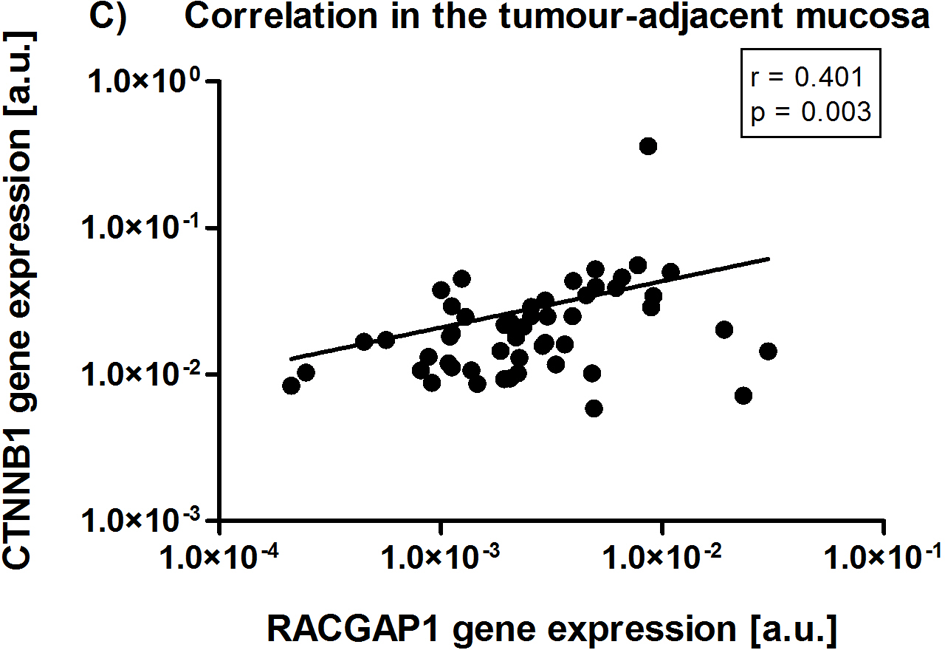

Supplement: Supplementary file 5 [file CAM4-5-516-s005.tif]
